# Antimicrobial Activity of Teat Antiseptic Formulations Based on Plant Extracts for Controlling Bovine Mastitis: In Vitro and In Vivo Evaluation

**DOI:** 10.3390/vetsci12040293

**Published:** 2025-03-21

**Authors:** Gabriel Michelutti do Nascimento, Romário Alves Rodrigues, Heloisa Cristina Brugnera, José Carlos Barbosa, Flavio Rubens Favaron, Gabriel Augusto Marques Rossi, Caio Roberto Soares de Bragança, Ruben Pablo Schocken-Iturrino, Fernando Antônio de Ávila, Marita Vedovelli Cardozo

**Affiliations:** 1 Department of Pathology, Reproduction and One Health, Sao Paulo State University—UNESP, Jaboticabal 14884-900, SP, Brazil; romario.a.rodrigues@unesp.br (R.A.R.); heloisabrugnera@gmail.com (H.C.B.); pablo@fcav.unesp.br (R.P.S.-I.); f.avila@unesp.br (F.A.d.Á.); 2 Department of Exact Sciences, Sao Paulo State University—UNESP, Jaboticabal 14884-900, SP, Brazil; jc.barbosa@unesp.br; 3 EquineBasic Company, Belo Horizonte 31110-170, MG, Brazil; zoo2008@hotmail.com; 4 Department of Veterinary Medicine, University of Vila Velha, Vila Velha 29102-770, ES, Brazil; gabriel.rossi@uvv.br; 5 Department of Biomedical and Health Sciences, Minas Gerais State University—UEMG, Passos 37900-106, MG, Brazil; caio.braganca@uemg.br (C.R.S.d.B.); marita.vedovelli@unesp.br (M.V.C.)

**Keywords:** herbal medicines, phytotherapeutics, herbal therapy, herbal teat dip

## Abstract

Pre- and post-milking teat antisepsis is one of the most effective methods for reducing the incidence of mastitis. Plant extracts have proven effective in reducing microorganisms, providing a natural alternative for antisepsis, along with additional benefits for teat health. This study aimed to evaluate the antimicrobial and antiseptic effects of two newly formulated products, used as pre-dipping and post-dipping agents, respectively, based on plant extracts from papain (*Carica papaya*), aloe vera (*Aloe barbadensis*), andiroba (*Carapa guianensis*), copaiba (*Copaifera officinalis*), tea tree (*Melaleuca alternifolia*), and barbatimão (*Stryphnodendron barbatiman*). Minimum inhibitory concentration tests were performed in vitro, along with microbiological analyses of different bacterial groups and in vivo compliance assessments to evaluate the antimicrobial activity of the formulations, as well as the molecular identification of *Staphylococcus aureus*. The results demonstrated that the products were effective in vitro against the main pathogens that cause mastitis, including *Staphylococcus aureus*, *Streptococcus agalactiae*, and *Escherichia coli*. In the in vivo assays, similar performance was observed between the tested products and the control products, indicating that the plant-extract-based formulations, applied as pre-dipping and post-dipping agents, effectively reduced the microbial load on the teats. The effects were equivalent to those of hydrogen-peroxide- and iodine-based products, being able to reduce the total microbial load by more than 80%.

## Simple Summary

Bovine mastitis is one of the most significant diseases affecting dairy production, leading to economic losses and reduced milk quality. Using antiseptic solutions before and after milking (pre-dipping and post-dipping) is an effective strategy for preventing this condition. This study evaluated the antimicrobial activity of two newly formulated products containing plant extracts. Laboratory and field tests demonstrated that these formulations effectively reduced the microbial load on the teats, performing similarly to conventional hydrogen-peroxide- and iodine-based products. These findings suggest that plant-based products offer a natural and sustainable alternative for mastitis prevention, contributing to animal health and milk safety.

## 1. Introduction

Bacteria from the *Staphylococcus* and *Streptococcus* genera are among the primary causative agents of bovine mastitis, followed by coagulase-negative *Staphylococcus* (CNS) and *Escherichia coli* [1,2]. While these microorganisms are the most prevalent in mastitis cases, bacteria from the *Enterobacteriaceae* family also warrant attention. Genera such as *Escherichia* spp., *Shigella* spp., *Salmonella* spp., *Klebsiella* spp., *Proteus* spp., *Morganella* spp., *Yersinia* spp., *Enterobacter* spp., and *Serratia* spp. are noteworthy. Additionally, genera such as *Acinetobacter*, *Pasteurella*, *Pseudomonas*, *Arthrobacter*, *Bacillus*, *Enterococcus*, and *Serratia* may act as opportunistic agents and environmental pathogens in bovine mastitis [3,4].

The application of sanitization methods is highly effective in preventing mastitis, as these methods are capable of reducing microbial concentrations on animal skin and thereby aiding in the disease’s prevention. Among the most commonly employed sanitization techniques worldwide are pre- and post-milking dipping processes. This involves immersing the teats in antiseptic solutions before and after milking, which significantly reduces the microbial load on the animal’s teats, decreases the incidence of contagious and environmental mastitis, and acts as an emollient, enhancing the natural healing process of the teat skin post-milking [5].

For this reason, sanitizers are selected based on their antimicrobial properties, which are capable of reducing the microbial load on the animal’s teats. Generally, the most commonly used products are based on iodine, chlorine, and hydrogen peroxide. However, studies have highlighted the negative effects and the presence of these substances in food, along with their consequent impacts on human, animal, and environmental health, unlike plant-based compounds [6,7,8,9,10,11]. Thus, plant-extract-based formulations have emerged as one of the alternatives to mitigate these impacts, and several studies have investigated the therapeutic properties of these compounds [12].

Plant extracts are currently being studied for their anti-inflammatory, antioxidant, immunostimulatory, wound-healing, and antimicrobial properties. *Carica papaya*, for example, contains a wide spectrum of bioactive compounds such as alkaloids, tannins, phenolics, flavonoids, saponins, terpenoids, sugars, glycosides, amino acids, steroids, and the enzyme papain, which exhibit antioxidant, anti-cancer, and wound-healing properties [13]. Similarly, several studies highlight the benefits of different *Aloe vera* species, including *Aloe barbadensis*, which is widely used in the pharmaceutical industry due to its biological activities. The bioactive compounds in its composition possess antioxidant properties, accelerate wound healing, stimulate the immune system, and contribute to improved bovine health [14].

These effects are comparable to those of *Stryphnodendron barbatiman* (barbatimão), which has potent wound-healing properties due to its promotion of angiogenesis and re-epithelialization [15]. *Carapa guianensis* (andiroba), meanwhile, also demonstrates wound-healing, anti-inflammatory, and additional pharmacological effects, such as antitumor and antimicrobial activities, which are also being explored. It is noteworthy that andiroba oil can be used as a mosquito repellent due to the presence of limonoid agents, which could be a relevant factor in the formulation of antiseptic products [16]. Additionally, copaíba oil and *Melaleuca alternifolia* (tea tree) oil are known to exhibit antimicrobial and antifungal activities against pathogenic microorganisms and bovine mastitis strains [17,18]. Thus, extracts and their biomolecules have been extensively explored by the pharmaceutical and cosmetic industries due to their functional properties, low cost, and biodegradability. This potential highlights the importance of research on their molecular composition, pharmaceutical formulations, and applications in the animal and human production chains [19].

Therefore, plant extracts have shown significant potential to reduce the total microbial load following their application, suggesting that a formulation incorporating some of these compounds may exhibit effective bacterial reduction during the preparation of teats and the antisepsis process, a critical step in milking management. At the same time, they represent a viable alternative for the organic dairy industry, being a product of natural origin. Thus, the present conducted in vitro and in vivo assays to evaluate the antimicrobial and antiseptic effects of two newly formulated products, used as pre-dipping and post-dipping, respectively, based on plant extracts from papain (*Carica papaya*), Aloe vera (*Aloe barbadensis*), andiroba (*Carapa guianesis),* copaiba (*Coparifera officinalis*), tea tree (*Melaleuca alternifolia*), and barbatimão (*Stryphnodendron barbatiman*).

## 2. Materials and Methods

### 2.1. In Vitro Testing

For the in vitro tests, the minimum inhibitory concentration (MIC) and minimum bactericidal concentration (MBC) assays were performed based on adaptations of the CLSI protocol with another study [20,21] to evaluate the pre-dipping formulation (Product 1) and the post-dipping formulation (Product 2).

After inoculation on an enrichment medium, a 0.85% saline suspension was prepared for each bacterial strain and standardized using a spectrophotometer at OD600, corresponding to 0.5 McFarland. Under aseptic conditions, a pilot test was performed to confirm the total bacterial count (CFU/mL) of the suspension using the spread plating procedure. Subsequently, the bacterial suspension was adjusted in Mueller–Hinton broth (Kasvi, Brazil) so that the inoculum on the ELISA plate corresponded to 5 × 10^5^ CFU/mL. The assay was performed in triplicate for each bacterial strain, including *Staphylococcus aureus* (ATCC 25923), *Streptococcus agalactiae* (ATCC 13813), and *Escherichia coli* (ATCC 25922).

To evaluate the effect of the combined plant extracts, the formulations were subjected to two-fold serial dilutions in a 1:2 ratio; for this purpose, 150 µL of the formulations was transferred to a 96-well ELISA plate containing 150 µL of Mueller–Hinton broth, as well as the positive and negative controls for MIC. The results are expressed as a percentage, considering that there are no specific clinical breakpoints for plant-extract-based products. After incubation, the MIC was determined as the highest dilution that visually inhibited bacterial growth in the evaluated well.

This dilution, along with the preceding dilutions, was plated onto Mueller–Hinton agar to determine the MBC, defined as the lowest concentration of the tested compound capable of inhibiting 99% of bacterial growth.

### 2.2. In Vivo Testing

The in vivo tests were conducted to evaluate antimicrobial activity and product compliance. For this purpose, we sampled Girolando dairy cattle during the peak lactation period (between 30 and 60 days); the cattle were housed on two dairy farms in Jaboticabal, São Paulo, Brazil. The farms housed around 150 dairy cattle and none of them produced organic milk. Milking occurred inside a milking stall; the cows had their teats washed with water and dried with clean paper towels before attaching the teat cups and beginning the process.

The animals were divided into two groups to evaluate the protocols: treatment and control. In the treatment group, the animals received the test products under evaluation. The tested products were derived from plant extracts and had two formulations (Products 1 and 2), used for the pre-dipping and post-dipping antiseptic processes, respectively. The products began to be administered by dipping the teats of lactating cows before the start of the study, with the application of ‘Product 2’ starting one day prior (24 h) and ’Product 1’ before attaching the teat cup apparatus, leaving the product for 30 s before the milking process began. These patterns were repeated for every collection period. Meanwhile, in the control group, the formulations were based on hydrogen peroxide (Product 3) for the pre-dipping solution and iodine (Product 4) for the post-dipping solution, both of which are commonly used on dairy farms for the prevention of bovine mastitis. The composition of all the products evaluated during the experiment, as well as the concentration of each plant extract, is described in Table 1.

**Figure 1:**
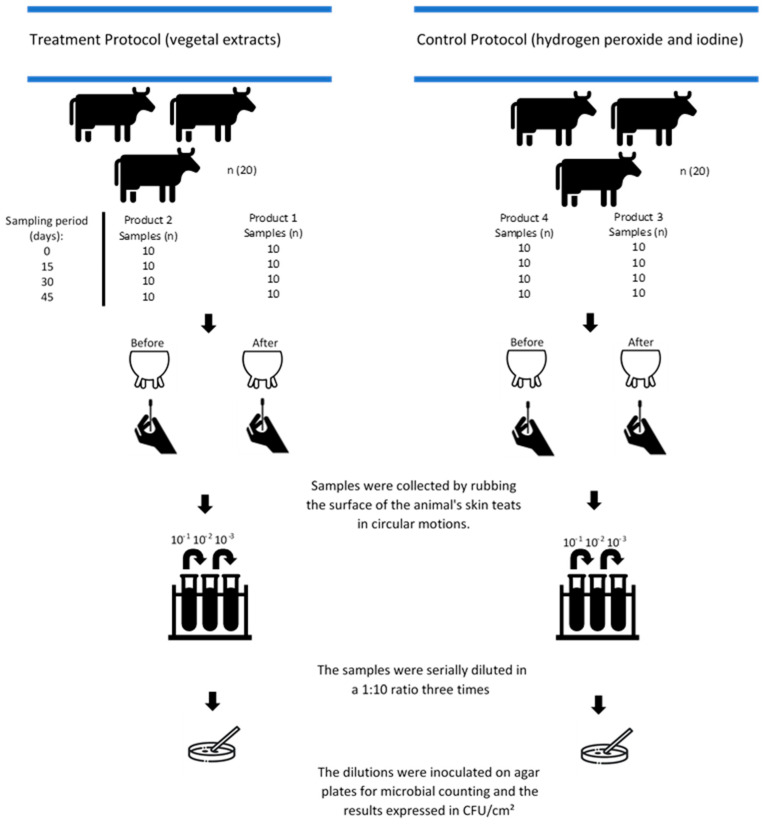
Sampling and processing scheme of the products for each period: plant extracts and conventional formulations tested on dairy cows before milking (Products 1 and 3, pre-dipping) and after milking (Products 2 and 4, post-dipping).

Samples were collected at four different time points, with a 15-day interval between them, on two dairy farms. At each time point, 20 animals were used per farm (10 in each group) throughout the experiment. Samples were collected before and after the application of the antiseptic product on each animal. The samples collected before teat antisepsis were considered when evaluating the post-dipping products, as these products were applied to the animals the day before (Products 2 and 4), after milking. The samples collected after the application of antiseptic products (Products 1 and 3) were considered when evaluating the pre-dipping products, collected before milking. Thus, at each time point, 40 samples were obtained per farm, with 10 samples for the evaluation of each product, totaling 320 samples at the end of the experiment. An illustrated scheme of the sampling and processing is displayed in Figure 1.

The samples were collected using a sterile swab rubbed on the surface of the teats of lactating cows in a pre-established area equivalent to 10 cm^2^. The swabs were then stored in test tubes containing 4.5 mL of bacteriological peptone at 0.1% and placed in sealed and refrigerated boxes. Subsequently, they were hermetically sealed in insulated containers with ice for microbiological and molecular analyses.

#### 2.2.1. Microbial Quantification

Starting from the collected sample, considered as the initial dilution (10^−1^), serial dilutions were performed at a 1:10 ratio up to 10^−3^ in 0.1% bacteriological peptone broth. Therefore, 0.5 mL of the homogenized initial sample was transferred to a tube containing 4.5 mL of sterile 0.1% bacteriological peptone, and this process was repeated successively to complete the three dilutions. The quantification of microorganisms was carried out by counting the colony-forming units (CFUs) per square centimeter, following the spread-plating method.

After homogenizing all dilutions, 0.1 mL aliquots from each dilution were transferred to Petri dishes containing Mannitol Salt Agar (Kasvi, Brazil), MacConkey Agar (Oxoid, UK), Azide-Blood Agar (Kasvi, Brazil), Nutrient Agar (Kasvi, Brazil), and Potato Dextrose Agar (Oxoid, UK) for the growth of staphylococci, enterobacteria, streptococci, total mesophilic aerobes, and total fungi and yeasts, respectively. The aliquots were then evenly spread on the surface of the culture medium using a cell spreader, and the media were incubated in a bacteriological incubator at 37 °C for up to 48 h. After incubation, the colony-forming units (CFUs) were quantified to assess the microbial concentration.

#### 2.2.2. Statistical Analysis

The experimental data were subjected to an analysis of variance through a non-inferiority test, using a split-plot design (2 treatments involving iodine + hydrogen peroxide and plant extracts × 4 periods), conducted using the SAS software (version 1.1.0.714) package and Agro-Estat program version 1.1.0.714 (2024).

Microbial count results were transformed using Log (x + 1) and statistically analyzed using a completely randomized design with a 2 × 4 factorial scheme (2 treatments and 4 time points). Comparisons between the different treatments were conducted using the general linear model (GLM) procedure. Significant differences were estimated based on the Tukey’s test analysis.

Suspected *Staphylococcus aureus* strains were selected for molecular analyses. For selection, *S. aureus* identification was performed based on the biochemical evaluation pro-vided by the culture medium used in the quantification step. Yellow colonies that proliferated on Mannitol Salt Agar (Kasvi, Brazil) with an indicator revealing mannitol fermentation and acid production were considered indicative of *S. aureus*, isolated, and chosen for molecular identification analysis.

Genomic DNA was extracted based on the chloroform protocol, with modifications. The samples were cultured in BHI broth (Kasvi, Brazil) and centrifuged at 8000 rpm for 5 min. The supernatant was discarded, and the pellet was resuspended in 700 μL of extraction buffer (160 mM Tris pH 8.0, 60 mM EDTA pH 8.0, 20 mM NaCl, 0.5% sodium dodecyl sulfate). The samples were homogenized and incubated at 65 °C for 40 min. Subsequently, 300 μL of 5 M potassium acetate was added, gently mixed, and incubated on ice for 30 min. Next, 600 μL of chloroform–isoamyl alcohol (24:1) was added, the samples were vortexed and centrifuged at 12,000 rpm for 10 min, and the supernatant was transferred to another tube. Then, 1 mL of absolute ethanol was added, and the samples were incubated at 4 °C overnight, followed by centrifugation at 12,000 rpm for 17 min to precipitate the DNA. Another wash with 70% ethanol was performed, with centrifugation at 12,000 rpm for 10 min. After the evaporation of the ethanol in a drying oven, the DNA was dissolved in 40 μL of TE buffer (10 mM Tris, pH 8.0, 1 mM EDTA), quantified using NanoDrop™ (Thermo Scientific, Waltham, MA, USA), and stored at –20 °C.

The identification of *S. aureus* was performed using the specific primer *cydB*-aureus with the following nucleotide sequence: Forward (F): CCCATTTGCTTGGTCTGTAGTA and Reverse (R): GTCCAGCCCATTTCTGGATTA. This primer set produces a PCR product of 432 base pairs. Further details, including the thermocycler settings for the PCR reaction and the complete protocol for the extraction, are available in another study using the same primers [22].

The reaction preparation was carried out using reagents from Cellco (Brazil), following the supplier’s instructions. The PCR Master Mix was prepared with the following final concentrations: 1X Reaction Buffer (10X), 200 µM dNTPs (10 mM), 1.5 U Taq DNA Polymerase (5 U/µL), 0.5 µM of the primers (10 µM), and ultrapure water to bring the total volume to 25 µL per reaction. The reaction mixture was then homogenized using a mini centrifuge and distributed into molecular tubes.

The samples were then subjected to the MasterCycler Nexus Thermocycler (Eppendorf, Darmstadt, Germany) under the following cycling conditions for the specific primer used: an initial denaturation at 95 °C for 3 min, followed by 30 cycles consisting of denaturation at 94 °C for 1 min, annealing at 57.5 °C for 1 min, and extension at 72 °C for 1 min. The final extension was at 72 °C for 7 min.

After the PCR reaction, 5 µL of loading buffer was added to each sample, which was then loaded onto a 1.5% agarose gel for electrophoresis. Following electrophoresis, the results were interpreted by visualizing the gel under ultraviolet light using a GelDoc XR+ Photodocumentation System (Bio-Rad, Hercules, CA, USA).

#### 2.2.3. Evaluation of Post-Dipping Product Compliance

The compliance evaluation was conducted exclusively on the post-dipping products (Product 1 and Product 2), concurrently with the aforementioned sample collection, with the aim of comparing the plant-extract-based formulation to the iodine-based control product. This in vivo macroscopic evaluation was conducted immediately after milking. The analytical methodology was based on another study [23]. A 5-point scoring system was employed to assess key characteristics, with scores ranging from 1 (least desirable) to 5 (most desirable). The evaluated parameters included color, dripping immediately after dipping, formation of a drop on the teat end, teat covered with a film, and evenness of teat coverage.

## 3. Results

### 3.1. In Vitro Testing

The interaction of all extracts, when combined in the pre-dipping formulation, achieved the highest efficiency, with MIC values as low as 0.097% of their initial concentration, indicating a strong interaction between all extracts in the formulation and a high capacity to reduce the microorganisms evaluated. The formulation of Product 1 showed lower efficacy against *Escherichia coli*, with an MBC value higher than 0.390%, which was greater than the values observed for the other two strains evaluated but still demonstrated effective results at very low initial concentrations. Product 2, a post-dipping formulation, demonstrated bactericidal activity against *Staphylococcus aureus*, which was not observed for the other tested species. However, the formulation was able to inhibit the multiplication of the other species, highlighting its bacteriostatic effect, with MICs of 50% for *Streptococcus agalactiae* and 0.390% for *Escherichia coli* (Table 2).

### 3.2. In Vivo Testing

#### 3.2.1. Microbial Quantification

Regarding microbial quantification, it was observed that both products were similarly effective in reducing microbial concentrations throughout the entire evaluation period, demonstrating the comparable antimicrobial activity of the plant extract product in relation to solutions commonly used for teat antisepsis. Furthermore, Product 1 showed lower counts for *Enterobacteriaceae*, *Streptococcus*, and mesophiles at T30, whereas Product 2 exhibited reduced average counts for *Staphylococcus* at both T0 and T30, as well as for *Enterobacteriaceae* at T30 (Table 3). However, the mesophilic group treated with Product 1 displayed higher counts compared to the hydrogen-peroxide-based control 45 days after treatment (T45), as shown in Table 3.

The average reduction in total microorganisms for both antiseptics at the end of the evaluation period was greater than 80%, according to a simple arithmetic mean, where the isolated reduction capacity of each farm, for each microbial group, was added up and divided by the number of collection periods. This confirms the results, with emphasis on pre-dipping being greater than or close to 90%. It is worth noting that the results of the plant-extract-based formulation were slightly superior to the control product for all evaluated microorganism groups, as demonstrated in Table 4.

#### 3.2.2. Statistical Analysis

The analyses revealed no statistically significant difference between the formulations, indicating that, across all of the collections and microbial groups analyzed, both treatments were equally effective in reducing microbial concentrations. Specific variations were observed; for instance, as shown in in Table 3, Product 1 was considerably more effective in reducing Enterobacteriaceae overall, particularly in the first sampling period (T0). Conversely, in the third sampling period (T30), Product 3 was more effective in reducing this same microbial group. Despite these variations, the overall average reduction for each microbial group remained similar, highlighting the equivalence in antiseptic efficacy between the control and plant extract treatments. These results are shown in Table 5 and Table 6.

The results presented in Table 6 indicate a similar antiseptic potential between the plant extract product and the control product, as both were equally effective in reducing all microbial agents evaluated in this study, with no statistically significant differences between them.

#### 3.2.3. Molecular Identification of *Staphylococcus aureus*

All evaluated samples tested negative for the identification of *Staphylococcus aureus* according to a PCR using the *cydB*-aureus primer.

#### 3.2.4. Evaluation of Post-Dipping Product Compliance

**Figure 2:**
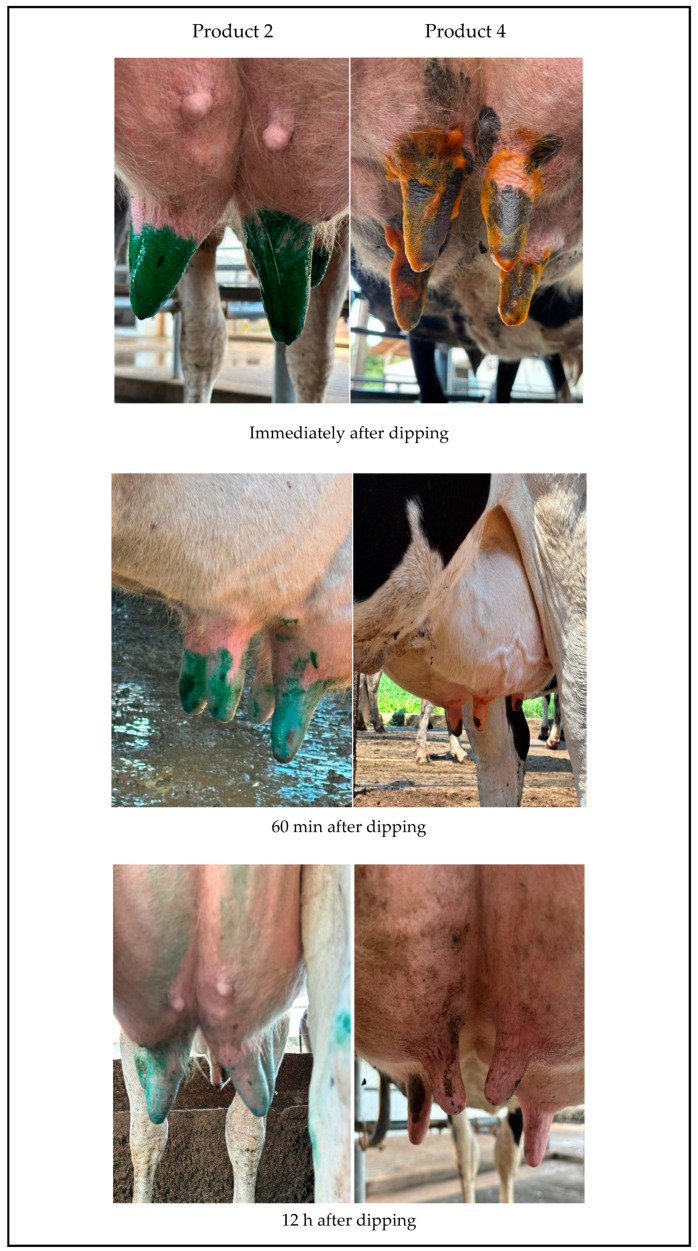
Evaluation of the conformity of post-dipping products: Product 2, based on plant extracts, and Product 4, based on iodine.

In the tested products, the color is bright immediately after teat dipping and remains well-defined for 10 min, gradually losing intensity over 60 min. Twelve hours later, the plant-extract-based product shows more visible residual color compared to the iodine-based product. For both products, the formation of a single stable drop is observed immediately after dipping, with a slower drop formation noted for the plant-extract-based product. Both products uniformly cover the teat, forming a film over the skin and teat; however, this coverage is more prominent for Product 2 (Figure 2, Table 7). According to the methodology used, the score for the plant-extract-based product is higher than that of the control product, which is commonly used on farms. It is worth highlighting that, macroscopically, throughout the sampling period, there was an absence of flies and an improvement was observed in the fissures and cracks in the nipples, as well as in the coloration and dryness conditions of the teats that were immersed in the plant-extract-based product.

## 4. Discussion

In the in vitro tests, the results demonstrated that the plant-extract-based products effectively eliminated and inhibited bacterial multiplication, with the formulation tested as a pre-dipping treatment showing the highest efficacy, as expected. Additionally, the plant extract formulations exhibited statistically equivalent performance in reducing the total microbial load on the teats of lactating cows compared to the control group formulations. The similar mean reduction values observed across all groups for both products highlight not only their comparable microbial reduction capacity but also their sustained long-term efficiency. Furthermore, the effectiveness of the dipping solution tested as a post-dipping treatment is attributed to its uniform and cost-effective composition, which provides adequate coverage of the teat canal and forms a protective film after milking.

Both formulations tested contain the components *Aloe barbadensis* leaf glycolic extract, *Carapa guianensis* seed oil, *Carica papaya* extract, *Copaifera officinalis* resin, *Melaleuca alternifolia* leaf oil, and *Stryphnodendron barbatiman* glycolic extract (Table 1). In the present study, a significant reduction in *Staphylococcus aureus*, followed by *S. agalactiae* and *E. coli*, was observed in the in vitro tests, demonstrating the antimicrobial activity of the plant-extract-based formulation designed for use as a pre-dipping agent (Product 1) at low concentrations, effectively eliminating microbial growth. Product 2, in turn, was used as a post-dipping agent; it exhibited bacteriostatic activity at higher concentrations, meaning it was only able to inhibit microbial proliferation, and its effectiveness was lower against *S. agalactiae* and *E. coli* (Table 2). The bacteriostatic effect of Product 2 can be attributed to the presence of *Copaifera officinalis* resin and *Melaleuca alternifolia* oil, as evidenced by previous studies demonstrating their activity against *Staphylococcus aureus* and *Staphylococcus saprophyticus*, respectively, with *Melaleuca alternifolia* oil being present at a higher concentration in the formulation of the post-dipping product [24,25,26,27].

Analyzing the results of both products, a better action against *S. aureus* and *S. agalactiae* was noted, which may be related to the ability of plant extracts and their bioactive compounds to act primarily against Gram-positive microorganisms, as previously observed in other studies [28,29]. Similarly, a study involving 45 medicinal plant extracts observed that *Streptococcus faecalis* was susceptible to most of them, including *Carica papaya*, which is present in the formulations tested in the current study, while *E. coli* demonstrated resistance to the majority of the extracts [30]. The oil of *Copaifera officinalis* also demonstrates better activity against Gram-positive microorganisms [31].

However, there are studies that support the antimicrobial effect in both bacterial groups, depending on the extracts analyzed. Aloe vera (*Aloe barbadensis*) leaf extracts have demonstrated such effects, including against the pathogens *Staphylococcus aureus* and *Escherichia coli*, in addition to exhibiting antifungal activity [32,33]. The oil of *Melaleuca alternifolia* also demonstrates activity against both bacterial groups, although it shows low efficacy against biofilm-forming microorganisms and yeasts [34,35]. Few studies support the antimicrobial effect of *Stryphnodendron barbatiman* and other similar species, as well as the seed oil of *Carapa guianensis*, and those that do often report activity only at high concentrations [36,37]. Additionally, papain, a bioactive compound derived from *Carica papaya*, efficiently disrupts biofilms formed by *Staphylococcus aureus* and *Staphylococcus epidermidis* and has the ability to enhance the efficacy of antimicrobial agents [38]. This finding is relevant for the development of formulations targeting microorganisms present in the udder and milk.

The results demonstrate the high antimicrobial activity of Product 1 and the lower efficiency of Product 2 in in vitro tests (Table 2). In spite of this, the in vivo results indicate the equivalence of both tested products with the control products (hydrogen peroxide and iodine) (Table 5 and Table 6). Product 1 was able to reduce nearly 90% of all evaluated microorganism groups, while Product 2 achieved a reduction of approximately 80% (Table 4). These results demonstrate that the combination of extracts in the tested formulations was effective in reducing all evaluated groups, showing a decrease in the counts of *Staphylococcus* spp., *Enterobacteriaceae*, *Streptococcus* spp., total mesophiles, fungi, and yeasts.

When evaluating and observing the action of the extracts individually, the antimicrobial response may vary depending on the tests performed and the microorganisms analyzed, which belong to different genera, including *Staphylococcus* spp., *Streptococcus* sp., and the *Enterobacteriaceae* family, as well as other mesophilic microorganisms, fungi, and yeasts. Most studies assess the activity of extracts against specific microbial species, and there is a lack of research focusing on their effects on broader bacterial and fungal groups [39,40,41,42]. When combined, these extracts may exhibit synergistic, non-synergistic, or additive effects on antimicrobial activity [43,44]. The in vitro results and the literature cited above are consistent with the in vivo findings of the present study, which seem to indicate the additive effects of the extracts on microorganisms belonging to different genera. This occurs when plant extracts and their metabolites result in the sum of individual effects, without synergism.

There were no significant statistical differences between the tested products and the control products. However, positive results were observed over the analyzed period, with Product 1 showing better counts for *Enterobacteriaceae*, *Streptococcus*, and mesophiles at T30, while Product 2 demonstrated lower average counts for *Staphylococcus* at T0 and T30, as well as for *Enterobacteriaceae* at T30 (Table 3). Although not statistically significant, the mesophilic group treated with Product 1 exhibited higher counts compared to the hydrogen-peroxide-based control 45 days after treatment (T45). A possible explanation for this finding is the formation of biofilms by different bacterial genera or mixed cultures within the mesophilic group, which may have increased their tolerance to the tested antiseptics. Biofilm formation is a critical factor in the development of resistance to antimicrobial agents. Nevertheless, plant extracts, essential oils, peptides, and other compounds have been shown to effectively interfere with biofilm production, demonstrating their efficacy in mitigating and reducing biofilm formation [45].

A study demonstrated that formulations containing plant extracts did not induce cytotoxicity in cells or adverse reactions in tissues, highlighting that they are an effective formulation for the healing process. This formulation included andiroba oil, copaiba oil, aloe vera, glycolic extract of *Stryphnodendron barbatiman*, tea tree oil, and glycolic extracts of *Carica papaya*, which are also present in the formulations tested in this study [46]. These findings align with our results, as Product 2 showed greater efficiency in the post-dipping product compliance assessment compared to the iodine control (Table 7 and Figure 2). Additionally, clinical observations demonstrated its efficacy in resolving nipple fissures and cracks, as well as improving teat color and dryness conditions (Figure 2, Table 7).

Pre-dipping products are used prior to milking and play a significant role in reducing the microbial load on teats, improving milk quality, and decreasing the risk of mastitis in animals. Bacterial colonization on the teat surface is one of the factors that increases the incidence of mastitis, with *Staphylococcus aureus* being the main pathogenic agent responsible for severe cases of mastitis [47]. In the present study, *Staphylococcus aureus* was not identified by PCR among the isolated *Staphylococcus* spp. Since it is a contagious agent, its absence can be expected, as it is associated with the presence of animals with intramammary infections and improper milking management. Therefore, environmental pathogens are more commonly found on teat surfaces and in the environments of lactating animals [48].

Post-dipping products primarily function to limit the colonization of microorganisms after milking, protect against pathogen entry, and assist in the closure of the teat sphincter to prevent new cases of mastitis. Due to the irritating properties of antiseptics commonly used in post-dipping, emollients are often added to improve teat condition. The tested product (Product 2) met the expected characteristics of a post-dipping product, which are attributed to the extracts in its composition. These extracts exhibit antioxidant, healing, immunological, and fly-repellent properties [49,50,51,52].

The control of bovine mastitis depends primarily on disease prevention rather than treatment after its onset. Additionally, there is not a single alternative to control this disease but rather a combination of measures that reduce the chances of bovine mastitis development and the associated damage. The use of antibiotics, herbal-based products, and other methods, when used individually, may not be as effective as a holistic approach; therefore, the conscious and combined use of all these measures together would be the ideal scenario. Additionally, the natural product, besides having antiseptic effects comparable to the control, would be economically more viable, making it a potential future alternative for dairy producers.

It is important to note a limitation of the MIC test, which was the use of an ATCC strain recommended for disk diffusion testing. The tested product does not fall within the parameters defined for conventional antibiotics; therefore, the methodology employed was adapted to meet the study’s objective.

Finally, the control of bovine mastitis primarily relies on the prevention of the disease rather than treatment after its onset. The use of herbal-based products, when applied individually, may not be as effective as when combined. Moreover, natural products, in addition to having antiseptic effects comparable to the control, could be more economically viable, making them a potential alternative for dairy producers.

## 5. Conclusions

The results of this study confirm the similarity between plant-extract-based products and those commonly used as pre- and post-dipping agents. These findings highlight their ability to reduce groups of microorganisms frequently associated with bovine mastitis, presenting them as viable alternatives for teat preparation in milking management.

## Figures and Tables

**Table 1:** Composition of antiseptic products used for pre-dipping and post-dipping: plant extracts (Products 1 and 2) and conventional formulations (Products 3 and 4) in percentages.

Product 1 * Pre-Dipping	Product 2 * Post-Dipping	Product 3 ** Pre-Dipping	Product 4 ** Post-Dipping
*Aloe barbadensis* leaf glycolic extract 2.5%	*Aloe barbadensis* leaf glycolic extract 2.5%	Hydrogen peroxide	Polyvynilpyrrolidone
E.D.T.A. 0.1%	Green dye 0.1%	Etidronic acid	Anionic surfactant
*Carapa guianensis* seed oil 2.5%	*Carapa guianensis* seed oil 2.5%	Decyl polyglucoside	Thickener
*Carica papaya* fruit extract 3.2%	*Carica papaya* fruit extract 3.2%	Benzalkonium chloride	Glycerin
*Copaifera officinalis* resin 0.2%	*Copaifera officinalis* resin 0.2%	Nanohydrate	Deonized water
Cosmoguard SL 0.5%	Glycerin 10%	Glycerin	
*Melaleuca alternifolia* leaf oil 0.2%	*Melaleuca alternifolia* leaf oil 0.4%	Hydroxyethylcellulose	
Propylene glycol 10%	Hyaluronic acid 0.3%	Formaldehyde	
*Stryphnodendron barbatiman* glycolic extract 2%	*Stryphnodendron barbatiman* glycolic extract 2%	Sodium hydroxixe	
Sodium lauryl ether sulfate 20%	Xantham gum 0.7%	Green HMC	
Deionized water 58.8%	Deionized water 77.8%	Deionized water	

* Treatment product (EquineBasic Profilacto^®^ pre-dipping and EquineBasic Profilacto^®^ post-dipping). ** Control product (Launer Biosani^®^ pre-dipping and Launer Forte Masty^®^ post-dipping).

**Table 2:** Results for minimum inhibitory concentration (MIC) and minimum bactericidal concentration (MBC), expressed as the dilution percentages of the tested formulations (Product 1 and Product 2) based on plant extracts against the bacterial species *Staphylococcus aureus, Streptococcus agalactiae*, and *Escherichia coli*.

Serial Dilution (%)	Test Product	*Staphylococcus aureus*		*Streptococcus agalactiae*		*Escherichia coli*	
		MIC	MBC	MIC	MBC	MIC	MBC
0	1	-	-	-	-	-	-
	2	-	-	-	- **	-	- **
50	1	-	-	-	-	-	-
	2	-	-	- *	+	-	+
25	1	-	-	-	-	-	-
	2	-	-	+	+	-	+
12.5	1	-	-	-	-	-	-
	2	-	-	+	+	- *	+
6.25	1	-	-	-	-	-	-
	2	-	-	+	+	+	+
3.125	1	-	-	-	-	-	-
	2	-	-	+	+	+	+
1.562	1	-	-	-	-	-	-
	2	-	-	+	+	+	+
0.781	1	-	-	-	-	-	-
	2	- *	- **	+	+	+	+
0.390	1	-	-	-	-	-	- **
	2	+	+	+	+	+	+
0.195	1	-	-	-	-	-	+
	2	+	+	+	+	+	+
0.097	1	- *	- **	- *	-	- *	+
	2	+	+	+	+	+	+

(-) = Growth inhibited. (+) = Growth. (*) The last concentration at which the MIC was observed, defined as the lowest concentration that visibly inhibits microbial growth. (**) The last concentration at which the MBC was observed, defined as the lowest concentration that results in 99% total microbial death. Cells highlighted in blue represent the last concentration with bactericidal activity, characterized by the equivalence of MIC and MBC. Cells highlighted in orange represent the concentration with bacteriostatic activity, where the MIC is lower than the MBC, indicating growth inhibition without complete microbial elimination. The reference strains used as a quality control responded within the acceptable limits.

**Table 3:** Mean decimal-scale counts of viable staphylococci, *Enterobacteriaceae*, streptococci, total mesophiles, and total fungi and yeasts according to the tested products, across all sampling time periods.

		**Treatment Group (CFU/cm^2^)**				
Time points (days)	Products	*Staphylococcus* spp.	*Enterobacteriaceae*	*Streptococcus* spp.	Total mesophiles	Total fungi and yeasts
0	Product 1	10^1^	10^1^	10^1^	10^2^	10^2^
	Product 2	10^1^	10^2^	10^2^	10^2^	10^3^
15	Product 1	10^1^	10^1^	10^1^	10^2^	10^2^
	Product 2	10^2^	10^1^	10^1^	10^3^	10^3^
30	Product 1	10^2^	10^1^	10^1^	10^2^	10^2^
	Product 2	10^3^	10^2^	10^2^	10^4^	10^4^
45	Product 1	10^1^	10^1^	10^1^	10^3^	10^2^
	Product 2	10^2^	10^1^	10^3^	10^3^	10^3^
		**Control Group (CFU/cm^2^)**				
Time points (days)	Products	*Staphylococcus* spp.	*Enterobacteriaceae*	*Streptococcus* spp.	Total mesophiles	Total fungi and yeasts
0	Product 3	10^1^	10^1^	10^1^	10^2^	10^2^
	Product 4	10^2^	10^2^	10^1^	10^2^	10^3^
15	Product 3	10^1^	10^1^	10^1^	10^2^	10^2^
	Product 4	10^2^	10^1^	10^1^	10^3^	10^3^
30	Product 3	10^2^	10^2^	10^2^	10^3^	10^2^
	Product 4	10^4^	10^4^	10^2^	10^4^	10^4^
45	Product 3	10^1^	10^1^	10^2^	10^2^	10^2^
	Product 4	10^2^	10^2^	10^3^	10^3^	10^3^

**Table 4:** Microbial reduction capacity (%) following the application of the tested products in pre-dipping (Products 1 and 3), including all microorganism groups.

			Reduction Capacity (%)			
Group	Sampling Periods	*Staphylococcus* spp.	*Enterobacteriaceae*	*Streptococcus* spp.	Total Mesophiles	Total Fungi and Yeasts
Treatment (Product 1)	0	92.0	91.5	92.2	81.2	90.8
	15	96.3	89.8	97.6	93.2	88.5
	30	92.4	91.0	72.8	93.4	95.2
	45	91.6	95.1	93.7	87.9	88.1
	Average	93.0	91.8	89.1	88.9	90.6
Control (Product 3)	0	81.7	53.1	96.1	80.7	92.1
	15	85.9	91.7	95.7	93.5	89.5
	30	97.7	97.6	71.3	80.8	92.3
	45	90.8	89.1	91.8	75.3	88.1
	Average	89.0	82.9	88.7	82.6	90.5

**Table 5:** The results of the Tukey’s test and the F-test were used to compare the similarity between protocols (plant-extract-based formulations vs. control products) across all microbial groups and sampling periods.

Statistical Analysis				Comparison Between Averages in Treatments							
Microbial Groups		Staphylococci		*Enterobacteriaceae*		Streptococci		Mesophilic Aerobes		Fungi and Yeast	
		Treatment	Control	Treatment	Control	Treatment	Control	Treatment	Control	Treatment	Control
Tukey’s Test ^1^		1.71 a	1.62 a	0.93 a	0.97 a	1.24 a	1.05 a	2.95 a	2.85 a	2.78 a	2.49 b
Periods				Comparison Between Averages In Sampling Periods (days)							
Farm 1	0	1.55 ab		0.83 a		0.79 b		1.95 e		1.95 c	
	15	1.86 ab		0.97 a		1.14 ab		3.20 abc		3.00 ab	
	30	1.43 b		0.81 a		0.79 b		2.51 d		2.66 b	
	45	1.38 b		0.95 a		1.04 ab		2.90 bcd		2.44 bc	
Farm 2	0	1.81 ab		0.87 a		1.24 ab		2.95 bcd		2.56 b	
	15	1.54 ab		1.25 a		1.31 ab		3.35 ab		2.62 b	
	30	1.66 ab		0.88 a		1.37 a		2.75 cd		2.56 b	
	45	2.10 a		1.07 a		1.48 a		3.60 a		3.30 a	
	F test ^2^	0.94 NS		0.72 NS		0.42 NS		0.63 NS		0.82 NS	

^1^ Values followed by the same letter display similarity in the reduction in the respective microbial group for each treatment, considering the total average from all sampling periods. Different letters indicate a difference in the reduction between treatments, albeit with no statistical significance when considering the total average reduction. ^2^ NS—non-signficant.

**Table 6:** F-test containing the mean reduction of every microbial group and every sampling period, displaying the statistical significance between the comparison of both protocols (treatment and control).

F-Test Results of the Total Average Reduction from All Microbial Groups Across All Sampling Time Periods					
Microbial Group	Group	Results	Group	Results	F-Test
*Staphylococcus* spp.	Control	1.02	Treatment	0.94	Non-significant
*Enterobacteriaceae*	Control	0.26	Treatment	1.7	Non-significant
*Streptococcus* spp.	Control	3.94	Treatment	2.42	Non-significant
Total mesophiles	Control	1.34	Treatment	0.64	Non-significant
Total fungi and yeasts	Control	9.28	Treatment	7.96	Non-significant

**Table 7:** Evaluation of the conformity of the post-dipping products (Products 2 and 4).

Parameters	Product 2	Product 4
Color		
	The color is bright and remains stable for 10 min. After 60 min, the color changed moderately. After 12 h, traces of the color persisted	The color is bright and remains stable for 10 min. After 60 min, the color changed moderately. After 12 h, traces of the color are slightly visible
Points (1 to 5)	4 points	3 points
Dripping immediately after dipping		
	No dripping after immersion (no more than one drop in the first minute)	No dripping after immersion (no more than one drop in the first minute)
Points (1 to 5)	5 points	5 points
Formation of a drop on the teat end		
	After a few minutes, a stable suspended drop is formed. After 60 min, the drop is no longer visible	After a few minutes, a stable suspended drop is formed. After 60 min, the drop is no longer visible
Points (1 to 5)	4 points	4 points
Teat covered with a film		
	It covers the teat skin with a uniform film in a single layer	It covers the teat skin with a uniform film, though slightly inconspicuous
Points (1 to 5)	5 points	4 points
Evenness of teat coverage		
	Covers uniformly.	Covers uniformly.
Points (1 to 5)	5 points	5 points
Total Points	23 points	21 points

## Data Availability

Data are contained within the article.
